# For a Comprehensive Health System

**DOI:** 10.1590/0104-1169.0000.2540

**Published:** 2015

**Authors:** Pedro Fredemir Palha

**Affiliations:** Pedro Fredemir Palha is Associate Editor of the Revista Latino-Americana de Enfermagem and Associado Professor of the, Escola de Enfermagem de Ribeirão Preto, Universidade de São Paulo, WHO Collaborating Centre for Nursing Research Development, Ribeirão Preto, SP, Brazil, palha@eerp.usp.br

**Figure:**
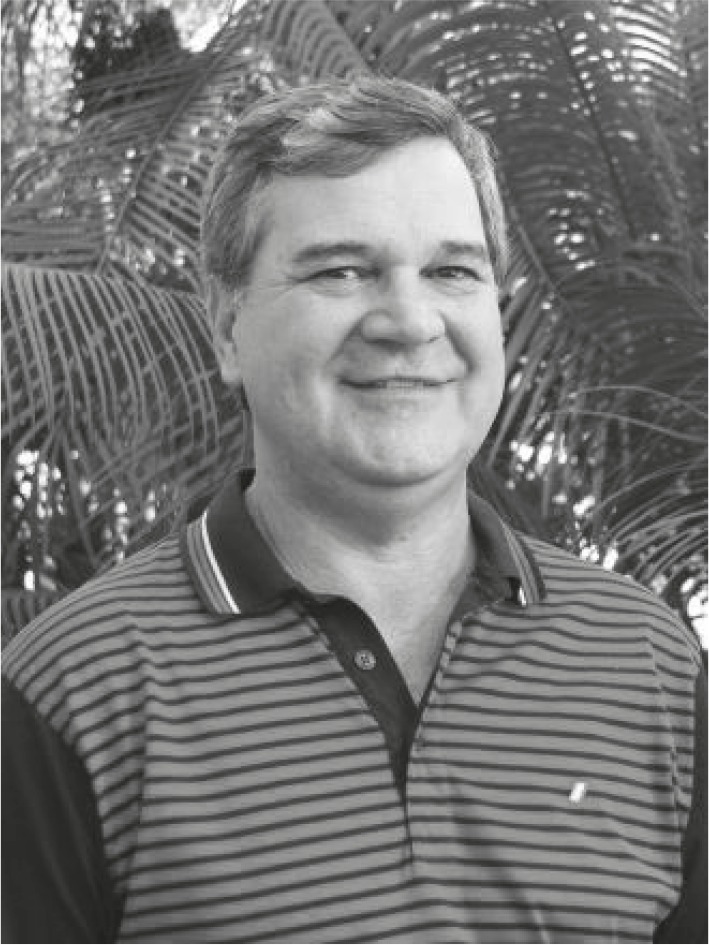

Providing quality, equality and universal access to all who, individually or collectively, seek a response to their needs are considered to be the great challenges in the health care area. In the current health context, countless countries of different levels of development have made efforts to guarantee meeting these objectives, differing from those of past decades; rapid ageing of the population, violence that is virtually an epidemic; the excessive burden of chronic conditions (communicable and non-communicable); accidents due to external causes; iatrogenesis of health care services; and service user safety, among others, all pose challenges for the health care and management field.

Despite strengthened public policies and positive discrimination in favor of the elderly, their increase in number and in life expectancy has been vertiginous in the last few decades. It is estimated that, in some countries, the population aged over 60 will represent 30% of the general population^(^ 1 ^)^. Such factors pose challenges for the different levels of care - primary, secondary and tertiary care - in which adults and the elderly make increasing use of care when faced with the individual characteristics of the ageing process. What is required, then, is appropriateness and innovation at the different levels of care, encouraging healthy ageing and reducing the risks that evolve from the physio-pathological processes of chronic diseases. The processed that generally affect quality of life in this population are those of non-communicable diseases which, when they become acute, increase early death despite the technical-scientific advances that have been made in the health care field. Such factors have had negative impacts from an economic point of view, from that of health care systems and of the families and individuals, aggravating social inequalities^(^ 2 ^)^.

As for violence, the World Health Organization has spread this concept globally, based on the idea of associating the intention to commit the act with actually committing it, irrespective of the result. Thus, health care services daily come across countless types of cases of violence, with immediate and latent repercussions on the most vulnerable groups in society, conditioning the health care services to adapt in order to tackle these processes. The consequences of violence affect different levels of the health care system differently, with increased treatment costs, as well as repercussions such as absenteeism and reduced productivity^(^ 3 ^)^. In addition to the immeasurable social and emotional costs for families and individuals affected by violence. 

Non Communicable Chronic Diseases (NCDs) have been placed on the agenda, requiring inter-sectorial dialogue and coordination to cope with their scale and the high social cost. Expressive differences- in gender, ethno-racial groups and socio-economic strata - within the same country, contribute in the way the burden of disease is distributed, especially among those who are socially vulnerable due to low income and schooling and difficulties accessing health care services. Thus, the context of the NCDs is one of the factors in health inequalities, requiring inter-sectorial action from the health care system aiming to reduce such inequalities^(^ 2 ^)^. 

I many countries, accidents due to external causes are the main public health care problems, being the principal cause of death in those aged under forty. They may occur suddenly, because of violence or an exogenous cause - injuries from automobile accidents, falls, homicide, attacks, drowning, poisoning, suicide, burns, injuries and natural disasters, as well as due to environmental circumstances. There is no way to calculate the social and economic impact on individuals and families^(^ 4 ^-^ 5 ^)^. As well as knowing the factors related to the event, the circumstances in which the trauma occurred also need to be recognized. This is an essential element in developing public policies for preventing, promoting or rehabilitating, which could reduce morbidity and mortality from trauma. Violence, then, is a challenge to organizing the health care system, from primary to tertiary care, and should be incorporated into the health care agenda. 

Situations involving iatrogenic events and user safety, related to the health care filed, is a complex phenomenon and has not been overcome, despite scientific advances with regards technological innovations related to health care and managing health care systems, benefitting health care professionals, managers and service users. Iatrogenic events are adverse or non-intentional events that considerably reduce the chances of user safety within the health care system. They have been approached in different ways, including economic, legal and health care service evaluation, among others. However, even if the circumstances can be recognized, it is difficult to evaluate the cause and effect involving this event and the health care system, given the legal and ethical difficulties involved in reporting occurrences. 

Iatrogenic events and user safety pose new challenges for health care systems. Associated with these events are the considerable number of health care professionals without job stability or in precarious employment relationships; this condition also encompasses limitations concerning qualitative aspects of human resources, inappropriate use of soft-hard and hard technology, as well as discontinuity of service user care. To this must also be added the lack of coordination between teaching, research and care, substantiated by the lack of instruments to qualify care, such as evidence based practice, guiding good practice in health care.

It should be recognized that the current care system lacks in-depth re-articulation between levels of care, despite advances in the health care sector in recent decades. Forming care networks, highlighted in the health care context, can potentially greatly reduce health care inequalities in the Brazilian geographical context, in which services are concentrated in the more economically developed regions calls into question the principles that guide the way care networks are organized, exacerbated by chronic underfinancing of the Brazilian health care system. 

There are, then, many challenges facing the Brazilian health care system. As for financing the system, this needs to be appropriate to and compatible with health care sector demands, ensuring the minimal funds needed for the sanitary thinking concerning universality, equality and sustainability in the sector. The following have become necessary: appropriate, defined roles of service providers, organized civil society participation, clearly defined tertiary sector participation in the care and management area and valuing health care professionals appropriately and qualitatively. Readjusting the health care model to meet the country's rapid demographic and epidemiological changes, incorporating care technologies that are appropriate to the health profile, dealing quickly with the epidemic of violence and promoting user care and safety at all levels of the health care system. Finally, it is evident that sectorial activities in the health care sector needs to be rapidly incorporated into strategic inter-sectorial actions as a way of facing different demands, for which the health care sector is not solely responsible, but also of integrated problem solving practices.
